# Nutrimiromics: Role of microRNAs and Nutrition in Modulating Inflammation and Chronic Diseases

**DOI:** 10.3390/nu9111168

**Published:** 2017-10-27

**Authors:** Bruna J. Quintanilha, Bruna Z. Reis, Graziela B. Silva Duarte, Silvia M. F. Cozzolino, Marcelo M. Rogero

**Affiliations:** 1 Nutritional Genomics and Inflammation Laboratory, Department of Nutrition, School of Public Health, University of São Paulo, 01246-904 São Paulo, Brazil; bquinta@hotmail.com; 2 Nutrition and Minerals Laboratory, Department of Food and Experimental Nutrition, University of São Paulo, 05508-000 São Paulo, Brazil; brunazreis@yahoo.com.br (B.Z.R.); graziela.biude@gmail.com (G.B.S.D.); smfcozzo@usp.br (S.M.F.C.); 3 Food Research Center (FoRC), 05508-000 São Paulo, Brazil

**Keywords:** microRNA, nutrients, inflammation, epigenetic

## Abstract

Nutrimiromics studies the influence of the diet on the modification of gene expression due to epigenetic processes related to microRNAs (miRNAs), which may affect the risk for the development of chronic diseases. miRNAs are a class of non-coding endogenous RNA molecules that are usually involved in post-transcriptional gene silencing by inducing mRNA degradation or translational repression by binding to a target messenger RNA. They can be controlled by environmental and dietary factors, particularly by isolated nutrients or bioactive compounds, indicating that diet manipulation may hold promise as a therapeutic approach in modulating the risk of chronic diseases. This review summarizes the evidence regarding the influence of nutrients and bioactive compounds on the expression of miRNAs related to inflammation and chronic disease in several models (cell culture, animal models, and human trials).

## 1. Introduction

Nutrigenomic refers to the study of interactions between nutrients and food components with the human genome, evaluating how gene expression and metabolic functions can be affected by feeding [1]. Nutrimiromics studies the influence of the diet on the modification of gene expression due to epigenetic processes related to microRNAs (miRNAs), which may affect the risk for the development of chronic diseases.

Considered as important regulators of gene expression, controlling both physiological and pathological processes, miRNAs are a class of non-coding endogenous RNA molecules (18–25 nucleotides in length). These molecules are usually involved in post-transcriptional gene silencing by inducing mRNA degradation or translational repression by binding to a target messenger RNA (mRNA) [2]. A single miRNA has the ability to regulate hundreds of mRNAs, however, not all miRNAs incur translational repression. Some miRNAs can activate the translation of proteins, control chromatin structure by regulating histone modification, or even directly target genes with a low DNA methylation [3,4,5,6].

**Figure 1:**
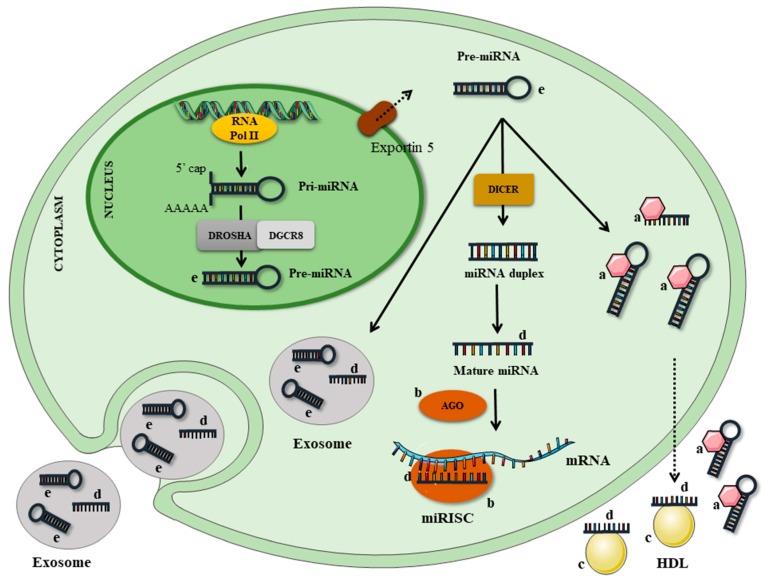
microRNA biogenesis and cellular release mechanisms. microRNAs (miRNA) is transcribed by RNA polymerase II from miRNA genes, first forming the ‘primary miRNA transcript’ (pri-miRNA), which is then cleaved by the DROSHA/DiGeorge syndrome critical region 8 (DGCR8) microprocessor complex to form the ‘miRNA precursor’ (pre-miRNA) (**letter e**). Pre-miRNA is then exported from the nucleus to the cytoplasm by exportin 5 and further processed by DICER1 to originate the mature miRNA (**letter d**). Mature miRNA is loaded into the miRNA-induced silencing complex (miRISC), which contains Argonaute (AGO) proteins (**letter b**), that targets mRNA by sequence complementary binding and mediates gene suppression by targeted mRNA degradation. The cellular release mechanisms include pre-miRNA or mature miRNA associated to RNA-binding proteins (**letter a**), such as Ago2 or their binding to high-density lipoproteins (HDL) (**letter c**). Furthermore, pre-miRNA or mature miRNA can be incorporated into small vesicles called exosomes, which are extracellular vesicles of endosomal origin that have emerged as key mediators of intercellular communication.

miRNAs are usually transcribed by RNA polymerase II from miRNA genes, first forming the ‘primary miRNA transcript’ (pri-miRNA). This transcript is then cleaved by microprocessor complex, which comprises the double-stranded RNase III enzyme DROSHA and its essential cofactor, the DiGeorge syndrome critical region 8 (DGCR8), originating a shorter sequence called the ‘miRNA precursor’ (pre-miRNA), which displays a hairpin-like secondary structure. The pre-miRNA is then exported to the cytoplasm and processed by DICER, a ribonuclease III enzyme that produces the mature miRNA, which is incorporated into an RNA-protein complex (RNA-Induced Silencing Complex—RISC). Under most conditions, the mature RISC represses gene expression post-transcriptionally by binding the 3′ untranslated regions (UTR) of specific mRNAs and mediating mRNA degradation, destabilization, or translational inhibition, according to sequence complementarity to the target [2,7,8] (Figure 1).

Significant amounts of miRNAs have been found, not only intracellularly, but also in extracellular human body fluids (e.g., serum, plasma, saliva, and urine), and have recently been shown to be associated with various pathological conditions [9].

There is much discussion about the origin and function of circulating miRNAs; it is not known with certainty if these molecules are products of cell excretion or if they undergo programmed secretion. However, some studies suggest that they have important functions, including the ability to modulate immune cells and the ability to transfer themselves to other cells or organs to exert tissue-specific roles [10,11]. These functions are mainly conferred by the presence of miRNA in vesicles, such as the exosomes (extracellular vesicles of endosomal origin), which have emerged as key mediators of intercellular communication [10]. Exosome-derived RNA can be transferred to other cells, and this genetic communication between cells may be occurring in the microenvironment, but could potentially also occur at a distance by the traffic of exosomes through the systemic circulation in a similar way to hormones. In fact, exosomes may be more effective in affecting a recipient cell if they deliver a specific miRNA that can modify recipient cell protein production and gene expression [12]. In addition to the exosomes, miRNAs can be coupled to a number of different structures: microvesicles, apoptotic bodies, lipoproteins (LDL or HDL), and ribonucleoprotein complexes (linked to Argonaut) [13] (Figure 1).

In addition to their regulation by proteins and mRNA, miRNAs can also be controlled by environmental and dietary factors [14,15,16,17]. Several studies have demonstrated that miRNAs can be regulated by feeding or even by isolated nutrients or bioactive compounds, indicating that diet manipulation may hold promise as a therapeutic approach to modulating the risk of chronic diseases [17,18,19,20].

Thus, this review will summarize the evidence regarding the influence of dietary components on the expression of miRNAs related to inflammation and chronic disease.

## 2. Inflammation and microRNA

The immune system has the major function of protecting the organism against pathogens, such as bacteria, viruses or parasites, tumoral cells, and endogenous stimuli, such as tissue damage. Once this response is activated, the immune system recruits a variety of cells and molecules that are capable of recognizing and eliminating or neutralizing the pathogen [21,22]. The immune system orchestrates responses through different mechanisms, including the adaptative and the innate immunity responses. The former is based on more specific receptors and provides a mechanism of response that depends on a variety of antigen receptors (immunoglobulins and T-cell receptors). Innate immunity constitutes the rapid response to an infection or a cellular injury and occurs in most organisms via its capacity to recognize molecular structures that are present in pathogens. The main receptors involved in innate immunity are the toll-like receptors (TLR) and the nucleotide-binding oligomerization-domain protein (NOD)-like receptors (NLRs).

Four types of defense mechanism are triggered during innate immune responses; anatomical, physiological, phagocytic, and inflammatory. Inflammation is an important component of the immune response, exerting beneficial effects by limiting responses to cellular or tissue damage [23,24,25]. The inflammatory response involves a variety of mediators that form a complex regulatory network that can be activated to provide protection against a type of aggression. During an ideal inflammatory response, neutralization of the offending insult should lead to the resolution of inflammation and ensure tissue homeostasis. Initially, the acute inflammatory process is triggered by infection or a tissue injury, and is characterized by an immediate recruitment of granulocytes to eliminate or neutralize the pathogen. This response normally results in tissue homeostasis, however, if the inflammatory stimuli persist, this results in chronic insults and excessive inflammation. The failure of inflammatory regulation mechanisms can prevent or delay the resolution of inflammation, leading to chronic inflammation. This chronic inflammatory response can also result from other causes, such as autoimmune responses (rheumatoid arthritis, psoriasis, lupus erythematosus, among others) and exogenous stimuli that can cause tissue damage [24,26,27,28,29]. However, the inflammatory process observed in obesity and the metabolic syndrome differs in many aspects from the classical inflammatory response. Metabolic inflammation or metainflammation—as proposed by Hotamisligil et al. [30]—does not involve the classic signs and symptoms of inflammation (pain, redness, tumor, and heat), and this inflammatory process manifests itself in a systemic way, being characterized by a chronic and low-intensity reaction. Metainflammation occurs as the consequence of the surplus of some nutrients and metabolites, and this process involves signaling pathways and molecules that are related to classic inflammation. The development of metainflammation has been attributed to a wide and integrated network of intracellular signaling pathways, in which two proteins are highlighted: the inhibitor of nuclear factor kappa-B kinase (IKK)-β and c-Jun N-terminal kinase (JNK)-1. IKK-β and JNK-1 promote the induction of the synthesis of inflammatory mediators in several cell types [30]. Activation of IKK-β and JNK-1 culminates in the activation of nuclear factor (NF)-κB and activator protein (AP)-1, respectively, which translocate to the cell nucleus and activate the transcription of several genes that are related to the inflammatory response, such as interleukin (IL)-1β, IL-6, and TNF-α. Some of these inflammatory mediators, such as IL-1β and TNF-α, bind to receptors on the plasma membrane of the cell, which results in the activation of intracellular signaling pathways that activate IKK-β and JNK-1, promoting the perpetuation of this inflammatory reaction, as implicated in conditions such as atherogenesis and insulin resistance [30,31].

The adipose tissue is considered to be an endocrine organ that, besides exerting the function of storing energy reserves in the form of triacylglycerols, produces and/or secretes several hormones and signaling molecules [32]. However, the main source of this systemic inflammatory response is the adipose tissue, which produces a variety of proinflammatory cytokines and chemokines, called adipokines, such as IL-1β, IL-6, TNF-α and monocyte chemotactic protein (MCP)-1. In this context, other tissues are also involved in inflammation, such as the liver, the pancreas, the hypothalamus, skeletal muscle, and pancreatic β cells [33,34,35].

In obese individuals, a greater synthesis of pro-inflammatory biomarkers occurs, such as MCP-1 (also called CCL2), soluble intercellular adhesion molecule (sICAM)-1, plasminogen activator inhibitor-1 (PAI)-1, TNF-α, IL-1 β, IL-6, and leptin. On the other hand, adipokines with anti-inflammatory actions, such as adiponectin, present reduced concentrations in these individuals. Leptin has the major function of controlling food intake and energy expenditure in the central nervous system. Some studies have shown that hyperleptinemia increases the inflammatory response by a largely unknown mechanism. Leptin purportedly triggers the production of reactive oxygen species (ROS) and increases chemotaxis, neutrophil activation, and the synthesis of proinflammatory cytokines, such as TNF-α, IL-6, and IL-12 [32,33,34,36,37,38].

An important finding that helped elucidate the cause of tissue inflammation in obesity was the observation that adipose tissue from obese humans is infiltrated with large numbers of macrophages. Obesity is a disease characterized by a constant infiltration of macrophages into the white adipose tissue, mainly in the visceral adipose tissue, and these cells play an important role in the production of pro-inflammatory factors. This process originates from the migration of peripheral blood monocytes to the visceral adipose tissue, which subsequently differentiates into macrophages. MCP-1 is the chemokine responsible for mediating this event since its expression is highly related to the amount of resident macrophages. In addition, MCP-1 positively correlates with adiposity. Gene expression and secretion of MCP-1 are stimulated by TNF-α, IL-6 and growth hormones in preadipocytes, and may be influenced by changes in adipokine secretion [39,40].

Macrophages and products secreted by adipocytes such as chemokines and cytokines, can activate endothelial cells, resulting in the production of a variety of adhesion molecules, chemokines, and cytokines, which in turn will direct leukocytes to underlying tissues and influence them to become fully active. Active macrophages can be classified into types M1 and M2. M1 macrophages act in the production of proinflammatory cytokines, such as IL-1β, IL-6, and TNF-α, which provoke the development of insulin resistance in adipocytes. On the other hand, the M2 phenotype represents those that were alternatively activated, characterized by the low expression of pro-inflammatory mediators and the high expression of IL-10 [27,39,41]. Several lines of evidence indicate that proinflammatory macrophages can cause insulin resistance. The proinflammatory factors derived from macrophages (TNF-α, IL-1 β, IL-6) are related to the development of insulin resistance. Furthermore, the insulin receptor substrate (IRS) proteins have an important role in insulin action, where the phosphorylation of IRS-1, induced by TNF-α or non-esterified fatty acids (NEFAs), for example, at serine residues can impair the capacity of this protein to engage in insulin-receptor signaling and alter insulin action. Additionally, the suppressor of cytokine signaling (SOCS) proteins, at the insulin-receptor substrate level, can also inhibit the action of insulin [30,42,43,44,45].

Two important signaling inflammatory pathways are involved in all these processes; namely the NF-κB and JNK pathways. The NF-κB pathway can be activated by various stimuli, such as TNF-α, lipopolysaccharides (LPS) and saturated fatty acids [33]. This signaling pathway involves the enzymatic complex IκB kinase (IKK), which induces the phosphorylation of Inhibitor-κB (IκB). The phosphorylation of IκB—the protein that inactivates the p50/p65 (NF-κB) heterodimer—results in its polyubiquitination, which, in turn, leads to its degradation mediated by the 26S proteasome. This degradation of IκB allows for NF-κB to translocate to the nucleus and to activate the transcription of several κB-dependent genes, such as those encoding proinflammatory cytokines, adhesion molecules, and chemokines. The JNK signaling pathway can be activated by stimuli, such as cytokines, fatty acids, and ROS. The active JNK pathway promotes the activation of the transcription factor activator protein 1 (AP-1), which is related to the gene expression of pro-inflammatory cytokines and may act directly on the insulin signaling pathway [45,46,47].

Several studies have shown that miRNAs are associated with inflammatory processes in metabolic diseases. During the inflammatory process, genes encoding proteins can be regulated at the transcriptional level and by miRNAs.The miRNA transcriptional process can be induced by inflammation, and these molecules rapidly become active as they do not need to be translated or translocated back into the nucleus to repress their targets. miRNAs are expressed in different cell types, have distinctive functions and their target proteins can be involved in the regulation of inflammatory response. Some proteins, for example, those induced during the inflammatory response can regulate the processing of miRNAs. On the other hand, miRNAs can also regulate several mechanisms that are involved in the initiation and resolution of inflammation, such as oxidative stress, macrophage activation, adipogenesis, and others [48,49,50,51].

In vitro studies show that some miRNAs, such as miR-20a, miR-17, and miR-106a, can contribute to macrophage infiltration in adipose tissue. These miRNAs stimulate macrophage infiltration by inhibiting the gene expression of signal-regulatory protein α (SIRP-α) [52]. In the subcutaneous tissue of animals, it has been observed that the expressions of miR-26b and miR-155 are also associated with the number of macrophages that are infiltrated in adipocytes [53]. In the vascular wall, miR-424 miR-155, miR-503, and miR-222 can contribute to the differentiation of monocytes into macrophages, unlike the miR-20a, miR-106a, and miR-17, which prevent this mechanism by suppressing the transcriptor factor acute myeloid leukemia-1 (AML-1) and downregulating macrophage-colony-stimulating factor receptor [51].

miR-21 is an important miRNA involved in inflammatory mechanisms. Studies in vitro using MCF-10A cells showed that the signal transducer and activator of transcription 3 (STAT3) activates directly the transcription of miR-21 during a cellular transformation process. Targets of miR-21, like the phosphatase and tensin homolog (PTEN), when its expression is inhibited, results in NF-κB activation, which is necessary to maintain this transformation process [54]. Human PBMCs treated with LPS observed an upregulation of miR-21 and a lower protein programmed cell death 4 (PDCD4) expression, which is a proinflammatory tumor suppressor and known target of miR-21. In RAW264.7 cells, it was observed that PDCD4 can inhibit the IL-10 mRNA translation, while the miR-21 has anti-inflammatory effects by increasing the production of IL-10 [55]. Das et al. [56] evaluated the role of miR-21 in the regulation of efferocytosis-mediated suppression of innate immune response in monocyte-derived macrophages. These authors verified the central role of miR-21 in the efferocytosis-dependent resolution of wound inflammation, since miR-21 downregulates proinflammatory response via blocking the TNF-a–NF-kB pathway, and upregulates anti-inflammatory IL-10 via the AP-1 pathway.

The miR-125 family has been associated with immune responses [57]. Blood samples of patients with systemic lupus erythematosus (SLE) and normal controls were collected and PBMCs were isolated for miR-125a expression analyses. It observed a lower expression of miR-125 in SLE patients as compared to controls and an increased expression of its target Kruppel-like factor 13 (KFL13). When miR-125a is overexpressed in T cells, there is a reduction in the expression of KFL13 and the chemokine RANTES (regulated on activation, normal T-cell expressed and secreted), or also known as CCL5. Thus, it is suggested that the overexpression of miR-125a can inhibit the levels of RANTES by controlling the expression of its target gene KFL13 [58]. The levels of this miR were found downregulated in peripheral CD4^+^ T cell of patients with Crohn´s disease. It seems that miR-125a is important in the regulation of T cell functions by suppressing the expression of effector T cell factors [59,60].

Another miR that seems to be involved in some inflammatory process is the miR-378. Xu et al. [61] evaluated the expression of miR-378 in human pre-adipocytes cells that were induced to differentiation. These cells were treated with 10 ng/mL of TNF-α, 30 ng/mL of leptin, 30 ng/mL of IL-6, or 60 ng/mL of resistin. Expression of miR-378 was found to be significantly elevated after TNF-α, IL-6, and leptin stimulation, whose result indicates that miR-378 probably is a novel mediator in the molecular mechanisms related to insulin resistance and obesity. Also, miR-378 has important roles in adipogenesis. Since miR-378a-3p expression is up-regulated during the differentiation of 3T3-L1 preadipocytes, as well as in adipose tissues of high fat diet-induced obese mice. It should be noted that miR-378a promotes adipogenesis by targeting mitogen-activated protein kinase 1 (MAPK1) [62]. Adiponectin expression in 3T3-L1 cells, an anti-inflammatory adipokine, can be modulated by miR-378 via the 3´UTR sequence-binding site since the expression levels of adiponectin and mirR-378 were negatively well correlated in these cells after being treated with TNFα [63].

Other miRNAs can regulate the secretion of inflammatory mediators. In cultured preadipocytes, let-7a/d, miR-193a/b, miR-145, and miR-26a can affect the secretion of CCL-2 and TNF-α. In contrast, miR-325 and miR-99a obtained from the subcutaneous and omental white adipose tissue of Caucasian individuals present a negative correlation with the secretion of IL-6 [53]. Chou et al. [64] investigated the effect of miR-221 on adipose tissue-derived mesenchymal stem cells that were obtained from obese women. These cells were isolated and induced to differentiation. During this process, miR-221 was found to be down-regulated and, in a second experiment, a positive correlation was observed between the decrease in miR-221 gene expression and the increase of TNF-α.

With regard to coronary artery disease (CAD), in the peripheral blood mononuclear cell (PBMC) of patients with stable and unstable angina pectoris, miR-147 was reportedly decreased 4-fold when compared to a control group. This miRNA can alter the inflammatory profile of immune cells by repressing TNF-α and IL-6 [65,66]. miR-143 and miR-145 are both highly expressed in smooth muscle cells (SMC) of normal arteries and down-regulated following vascular injury. Animals deficient in miR 143/145 have a lower blood pressure and thinner medial layer arteries, suggesting that these miRs are involved in the SMC homeostasis [67,68]. In arterial wall, circulating monocytes will adhere and infiltrate into the vascular intima. Some miRNAs such as miR-125b and miR-146a can contribute in the beginning of this inflammatory process, and others like miR-10a, miR-124a, miR-221/222 can promote anti-inflammatory effects [69,70,71,72]. In ApoE^−/−^ mice, the inhibition of the proathero-miR-342-5p reduced atherosclerotic plaque development in the aorta of these animals [67]. Furthermore, endothelial senescence is controlled by miR-217 and miR-34a. This mechanism is mediated by the inhibition of sirtuin 1 (SIRT1), a direct target of miR-34a and miR-217, inducing cellular senescence [73]. The expression of some miRNAs related to the NF-κB pathway can be induced in chronic inflammation conditions. miR-132, in differentiated primary adipocytes can contribute not only to macrophage infiltration, but also to the activation of the NF-κB signaling pathway, and increase the production of pro-inflammatory cytokines, such as IL-8 and CCl-2. Another miRNA that is associated with the NF-κB via is miR-497, which affects this pathway by a negative feedback mechanism. This miRNA can inhibit IKK-β and consequently reduce the phosphorylation of the inhibitor of NF-κB (IκB), preventing NF-κB translocation to the nucleus and its activation, resulting in a lower production of pro-inflammatory cytokines and an improvement in the chronic inflammatory process [74,75,76]. In contrast, miR-155 and miR-146a have an opposing effect on this transcriptional factor. miR-155 can be highly expressed in macrophages, monocytes, and myeloid cells in the presence of pro-inflammatory cytokines, such as IFN-γ and IFN-β. Studies have shown that miR-155 upregulation and the NF-κB activation are positively correlated [77,78]. This pro-inflammatory miRNA targets two negative regulators of inflammation, the suppressor of cytokine signaling 1 (SOCS1) and SHIP1. The repression of these regulators can stimulate the expression of inflammatory cytokines and interferon response in primary macrophages and dendritic cells obtained from mice, and this process occurs via miR-155 [79,80]. Additionally, this miRNA is important for a variety of inflammatory mediators since its upregulation has been observed following stimuli with molecules including cytokine IFN-β and Toll-like receptors ligands through myeloid differentiation factor 88 (MyD88), in bone marrow cells [81].

miRNA-146a, unlike miR-155, is considered to be a negative regulator of immune responses and was the first miRNA to be associated with TLR signaling. The importance of this miR in the inflammatory response is associated with its targets, TNF receptor–associated factor 6 (TRAF6) and IL-1 receptor-associated kinase 1 (IRAK-1), key molecules in the TLR4/NF-κB pathway [48,77]. This negative regulation occurs in the following sequence; the miR-146a gene is upregulated through activation of NF-κB and can prevent the production of signal transducer proteins. Pre-synthesized TRAF6 and IRAK1 proteins continue to transduce signals, and the action of miR-146a can be delayed causing a downregulation of inflammatory signaling cascades. After this process, a gradual downregulation of the inflammatory signaling cascades is observed in innate immune cells [48]. Boldin et al. [82] observed that, in mice in which the miR-146 gene was deleted, treatment with a sublethal injection of LPS significantly increased the production of pro-inflammatory cytokines IL-6, IL-1β, and TNF-α.

After the discovery of the importance of the miRNAs in several biological processes, such as in the inflammatory process mentioned above, many studies have been developed for a better understanding of their functions and interactions in the human organism. In the area of nutrition, nutritional genomics emerged with the objective of understanding the effects and interactions of nutrients and bioactive compounds with the genome. The search for new biomarkers for the evaluation or the discovery of new functions of a particular dietary component and the possible effects of a nutritional intervention contributed to the advances in the research in this area, and miRNAs are included in this context.

## 3. microRNA, Nutrients, Inflammation and Chronic Diseases

Few studies have sought to evaluate the effects of a nutrient or bioactive compound on the expression of a particular miRNA in a specific human health condition and vice versa. Given the impact of the inflammatory response in chronic diseases and the role of certain miRNAs in several steps of this process, it is important to evaluate which dietary components can modulate this response and how.

In this review, we summarize some of the studies that have evaluated the influence of nutrients and bioactive compounds on miRNA expression in several models (cell culture, animal models, and human trials).

### 3.1. Resveratrol

Resveratrol (3,5,4′-trihydroxystilbene) is a phytoalexin composed of two phenolic rings that are connected by a double bond; this bioactive compound is found in grapes, berries, peanuts and other plants. Resveratrol exists in two isoforms; trans-resveratrol and cis-resveratrol, where the trans-isomer is the more stable form. Many studies have reported on the antioxidant and anti-inflammatory effects of resveratrol, which confer anticancer and cardioprotective functions to this polyphenol [83,84,85,86,87].

The anti-inflammatory mechanisms attributed to resveratrol include a reduction in proinflammatory lipid mediators by inhibition of cyclooxygenase 1 and 2 (COX-1 and -2), and the reduction of TNF-induced translocation of the p65 subunit of NF-κB and reporter gene transcription, which in turn, decrease the expressions of pro-inflammatory cytokines, such as TNF-alfa, IL-1 beta, IL-6 [83]. Additionally, resveratrol can attenuate the activation of JNK and its upstream kinase mitogen-activated protein (MEK), which may explain the mechanism of suppression of AP-1. Tili et al. [15], in an experiment with human monocytes (THP-1), showed another possible pathway by which resveratrol may modulate AP-1 via epigenetic mechanisms. THP-1 cells were incubated with resveratrol (30 or 50 µM) for 14 h. As a result, resveratrol upregulated antiinflammatory miR-663, which directly targets two AP-1 factors (JunB and JunD), decreasing AP-1 activity. In contrast, pro-inflammatory miR-155 was down-regulated by resveratrol and by upregulated miR-663. Using other doses (25, 50, 100, and 200 mM) of resveratrol and treating the same lineage of cells (THP-1) for a long period (48 h). Song et al. [88] observed a higher expression of miR-Let7A as compared to non-treated cells. Moreover, resveratrol and/or miR-Let7A decreased mRNA levels of TNF-α and IL-6, and increased IL-10 after stimulation of these cells with LPS.

Li et al. [89] looked at the possible effects of resveratrol on pro-inflammatory miR-21 expression in human glioblastoma (U251) cells, and treated these cells with 10 or 50 µM resveratrol during 12 h. The phenolic compound decreased miR-21 expression and the inhibition of this miR led to a reduction in IkB phosphorylation and NF-kB activity. In vitro experiments usually use high concentrations of phenolic compounds, and do not simulate physiological plasma levels in vivo. However, Bigagli et al. [90] incubated macrophages (RAW 264.7) with concentrations of resveratrol, hydroxytyrosol, and oleuropein that were compatible with plasma physiological concentrations (5 and 10 µM). Hydroxytyrosol and Oleuropein are phenolic compounds found in olive oil, and, like resveratrol, have been associated with antioxidant and anti-inflammatory properties. When comparing these three compounds, just resveratrol and hydroxytyrosol (at 10 µM) down-regulated miR-146a. MiR-146a targets the transcription factor nuclear factor (erythroid-derived 2)-like 2 (Nfr2) responsible for inhibiting pro-inflammatory mediators; Nfr2 also was positively modulated by resveratrol and hydroxytyrosol after the stimulation of macrophages with LPS [90].

In one study in humans, researchers performed a randomized placebo-controlled study with 35 type-2 diabetic and hypertensive men, who received capsules containing placebo (maltodextrin), grape extract (laking resveratrol) (GE) and grape extract with over 8 mg of resveratrol (GE-RES) during one year. Modulation of miRNA involved in the inflammatory response was found in the group supplemented with GE-RES; miR-21, -181b, -663, and -30c2 were up-regulated and miR-155 and -34a were down-regulated after treatment with GE-RES, as compared to the control group, indicating that the modulation of miRNAs by resveratrol induces a buffering effect against physiological variation [91].

### 3.2. Saturated Fatty Acids (SFA)

High-fat diets (HFD), especially those rich in saturated fatty acids (SFA), have been linked to detrimental populational health outcomes. Studies have shown that HFD is implicated in dysbiosis, subclinical inflammation, and insulin resistance [92,93,94,95]. Notably, some SFA are considered to exert pro-inflammatory effects. In this context, lauric and palmitic acids can stimulate inflammatory responses in adipocytes, macrophages, beta-cells and other metabolic tissues, by activating the cell membrane TLR2 and TLR4, culminating in the expression of pro-inflammatory genes, such as TNF-α, IL-6, and IL-1 β [96,97,98]. Recent experiments reveal that SFA can modulate this pathway also by epigenetics mechanisms such as via microRNAs.

In vitro studies mainly show the effects of SFA on miRNA expression in cancer lineage cells [99,100,101]. However, Yang et al. [102] verified an increase of miR-29a expression after the treatment of human myocytes (L6 GLUT4myc) with palmitic acid. According to these authors, miR-29a is responsible for post-transcriptional inhibition of insulin receptor substrate (IRS)-1, significantly decreasing this protein’s concentration, and demonstrating a possible role of this miR in insulin resistance and diabetes. Takahashi et al. [103], using mouse cardiomyocytes (HL-1 cells), observed palmitate stimulation of miR-27b expression, suggesting an increased vulnerability to atrial arrhythmia.

### 3.3. Polyunsaturated Fatty Acids (PUFA)

Most studies have highlighted eicosapentaenoic (EPA) and docosahexaenoic (DHA) acids as the major fatty acids involved in the attenuation of the inflammatory response; for example, DHA reportedly inhibits NF-kB and JNK signaling through G protein-coupled receptor 120 (GPR120) by DHA [104,105]. Moreover, an in vitro study showed that the EPA and DHA-induced modulation of the expression of miRNAs involved in inflammation after the stimulation of macrophages (RAW 264.7) and epithelial (TIME) cells with LPS and pro-inflammatory cytokines (IL-1β, TNF-α and IFN-γ), and the treatment of these cells with DHA (C22:6n3) or arachidonic acid (AA, C20:4n6) [106]. As a result, the authors suggested an anti-inflammatory action of these PUFAs in the inflammatory milieu that was mediated by the downregulation of miR-146a, miR-146b, miR-21, miR-125a, and miR-155, which are related to the pro-inflammatory response that is triggered by NF-kB signaling [106].

PUFA can modulate the inflammatory response through lipid mediators that are synthesized from essential fatty acids, such as resolvins, thromboxanes, leukotrienes, and lipoxins. Some studies have observed the modulation of gene expression by these lipid mediators through miRNAs [107,108,109]. Wang et al. [110] demonstrated that leukotriene B4, synthesized from arachidonic acid, stimulated the inflammatory response by increasing MyD88 in mice macrophages via upregulation of miR-155 and -146b, which are responsible for SOCS-1 mRNA degradation and MyD88 inhibition.

Lipidomic strategies could contribute to identifying others fatty acids metabolites that are linked to inflammation resolution like resolvins, maresins, lipoxins, and protectins. These lipid mediators are PUFAs metabolites, and are called specialized proresolvins mediators (SPM) [111]. Li et al. [112] investigated the relation between miR-4661 and SPM in acute inflammation resolution. The authors verified a feedback mechanism in which miR-4661 increased after inflammation stimulation and up-regulated SPM that were responsible to decrease its expression.

Other studies have shown isolated effects of each one of these lipid mediators. Resolvins, for example, are DHA and EPA metabolites that originate in response to a tissue damage that contributes to inflammation homeostasis through down-regulation of NF-kB [111]. In this context, Recchiuti et al. [113] verified that miR-21, miR-146b, miR-142 family, miR-203, miR-208a, miR-219, and miR-302d that are temporally and differentially expressed in resolving exudates, and Resolvin D1, which is biosynthesized in resolution, regulates miR-21, miR-146b, miR-208a, and miR-219. Lipoxin A4 (LXA4) contributes to inflammation homeostasis through the modulation of chemotaxis, endothelial adherence, and transmigration of immune cells [114]. Codagnone et al. [114] observed one of the possible mechanism that LXA4 could promote those effects by miRNAs since miR-126-5p was significantly up-regulated by LXA4 in human umbilical vein endothelial cells.

Divergent effects were identified when other inflammatory pathways controlled by miRNA, such as PI3K/AKT, were analyzed. Vinciguerra et al. [16] showed that the treatment of hepatocytes with oleic acid led to a reduction in PTEN expression by up-regulating miR-21 via a direct effect of NF-kB p65 on the miR-21 promoter. Additionally, Chiu et al. [115], analyzing the possible effects of EPA on neovascularization, observed miR-221 inhibition by EPA, but not DHA, in human endothelial progenitor (hEPC) cells. This miRNA is linked to inhibition of the PI3K/AKT pathway [115].

Similarly to SFA, in vivo studies regarding the effects of UFA on miRNAs are very scarce. Zheng et al. [116], in an experiment with three-weeks years old rats fed on a mimic Western Diet for one week, compared miRNA expression in three groups: those fed on an omega-3 PUFA diet (composed of a mixture of EPA and DHA at a ratio of 1.5:1), an omega-6 PUFA diet (made with linoleic acid), and a control diet. The omega-3 PUFA diet group presented subcutaneous fat and pro-inflammatory cytokine reduction, when compared to the control group after 16 weeks of ingestion. rno-miR-19b-3p, -146b-5p, and -183-5p were down-regulated in the same group, but not in the omega-6 PUFA diet and control groups. After the prediction targets, the authors verified that these miRNAs are involved in the suppression of genes related to inflammatory pathways (TLR, MAPK, TGF-beta, RLR, NLR) [116].

In one experiment with humans, 30 healthy subjects consumed 30 g/day of almonds and nuts, which are food sources of polyunsaturated fatty acids (PUFA), for eight weeks. Alterations in the levels of 11 miRNAs were observed in the plasma. Among these, miR-328, miR-330-3p, miR-221, and miR-125a-5p had their expressions reduced, while miR-192, miR-486-5p, miR-19b, miR-106a, miR-130b, miR-18a, and miR-769-5p displayed increased levels after the intervention. According to the authors, miR-221 and miR-130b were associated with positive variations in plasma protein C-reactive (PCR) levels [17].

### 3.4. Curcumin

Curcumin, a member of the family of curcuminoid compounds, is a yellow polyphenol that is obtained from curcuma (*Curcuma longa* L.), also known as turmeric. This compound exerts important functions in the organism as a potent anti-oxidant, anti-diabetic, anti-cancer and anti-inflammatory agent, whose functions have been attributed to its hydroxyl and methoxy groups [33]. The intake of curcumin up to 8 g/day by humans does not show adverse effects [117]. With regard to the inflammatory response, curcumin can modulate the NF-κB pathway in vitro, thus preventing its activation by free radicals derived by TNF-α, phorbol 12-myristate 13-acetate (PMA) and hydrogen peroxide. Additionally, curcumin can inhibit the activation of JNK and transcription factor, AP-1, and also the phosphorylation and degradation of IκB-α [33,118]. Among several miRNAs involved in the inflammatory response, miR-155 is known as a key transcriptional regulator involved in this process. In a cell line of macrophages stimulated with LPS, the treatment with curcumin had a significant inhibitory effect on TNF-α and IL-6 production and on miR-155 expression, through the PI3K/AKT pathway. When a miR-155 mimic was transfected into a cell line and treated with curcumin, levels of cytokines (TNF-α and IL-6) were also reduced. In the same study, a sepsis mouse model was treated orally with curcumin (20 mg/kg) after LPS intraperitoneal injection. After curcumin treatment, miR-155 expression and AKT phosphorylation were reduced in samples of liver and kidney tissues [119].

Recent studies have shown that curcumin can modulate some miRNAs in some types of cancer cells through the inflammatory response. Kronski et al. [20] verified that the upregulation of miR-181b in breast cancer cells after treatment with curcumin is related to a down-modulation of pro-inflammatory cytokines CXCL1 and -2, causing an inhibitory effect on the metastatic process of these cells. Another study with breast stromal fibroblast demonstrated that the tumor suppressor p16^INK4A^ protein inhibits carcinogenic effects of these cells by repressing IL-6 expression and secretion, and this process is mediated by miR-146b-5p. This miR inhibits the expression of this cytokine at a specific sequence at IL-6 3´UTR. Treatment with curcumin may increase p16^INK4A^ and miR-146b-5p levels, and suppress IL-6 [120].

Besides inflammation, curcumin can also modulate some miRNAs related to other processes related to chronic diseases. In a study conducted in 3T3-L1 cells, miR-17-5p upregulates adipogenic differentiation and its inhibition repressed this process and the target, Tcf7l2. After treatment with curcumin, a down-regulation of the expression of miR-17-5p was observed together with an increase in the stimulus of its target in this cell line [121].

### 3.5. Quercetin

Quercetin is a bioactive compound found as aglycone (lipophilic) and is present in its glycosylated form (non-lipophilic) in citric fruits and apples. The role of quercetin in the inflammatory response is associated with the inhibition of ERK and JNK proteins, and its phosphorylated forms and the reduced synthesis of TNF-α generated from these proteins. In vitro, this polyphenol is able to inhibit the gene expressions of COX-2 and iNOS, and prevent the translocation of NF-κB to the nucleus, thereby attenuating the inflammatory response [33,122].

Studies evaluating the modulation of miRNAs related to inflammatory pathways by quercetin are scarce. Boesch-Saadatmandi et al. [123] observed that the hepatic levels of miR-125b and miR-122 were significantly higher in female mice that were fed on quercetin-enriched diets (2 mg/g), in comparison to the mice that received a control diet. miR-125b is known as a negative regulator of inflammation, while miR-122 is related to the regulation of lipid homeostasis. In another study, authors evaluated the effect of quercetin and its main metabolites on miR-155. In macrophages stimulated with LPS, treatment with quercetin and isorhamnetin down-regulated the expression of miR-155, possibly representing a mechanism by which this polyphenol may inhibit NF-κB activation and contribute to the attenuation of the inflammatory process [124].

### 3.6. Catechins

Catechins and their derivatives are thought to contribute to beneficial health effects by modulating miRNAs. There are several isomers of this compound; catechin, catechin gallate (CG), gallocatechin, gallocatechin gallate (GCG), epicatechin, epicatechin gallate (ECG), epigallocatechin, and epigallocatechin gallate (EGCG). EGCG is generally the most abundant and active polyphenol in green tea (*Camellia sinensis*), and is the only known polyphenol present in plasma in a large proportion (77–90%) in a free form after the intake of green tea [125,126].

In HepG2 cells, EGCG differentially represses the expression of five miRNAs (miR-30b*, miR-453, miR-520-e, miR-629, and miR-608) involved in inflammatory pathways, the peroxisome proliferators-activated receptors (PPAR) signaling pathway, the insulin signaling pathway, glycolysis and gluconeogenesis, oxidative phosphorylation, and glutathione metabolism [127]. EGCG up-regulated miR-let-7b expression not only in melanoma cell lines but also in metastatic melanoma tumors in vivo. miR-let-7b has several target genes related to tumor progression and seems to suppress melanoma tumor growth by activating the intercellular signaling pathway, cAMP/protein kinase A (PKA)/protein phosphatase 2A (PP2A) [128].

In human chondrocytes derived from osteoarthritic (OA) cartilage stimulated with IL-1β, EGCG differentially expressed 36 human miRNAs (19 were up-regulated and 17 were down-regulated). Furthermore, EGCG up-regulated miR-140-3p expression, which in turn was associated with the downregulation of aggrecanase-2 (or ADAMTS5), expression [19]. This effect may protect against early cartilage degradation, as aggrecan (a major component of the cartilage extracellular matrix) degradation by aggrecanases is a frequent event in OA progression [19]. It should be noted that most of these effects in cell cultures are observed at concentrations of EGCG of greater than 10 µM, which is not typically observed in vivo following tea consumption (usually < 0.5 μM). Even with the administration of 1600 mg of pure EGCG, the maximum plasma concentration observed is 7.4 µM [126].

### 3.7. Minerals

The mineral, selenium, is essential to human physiology since the amino acid selenocysteine is inserted into several selenoproteins with a wide range of functions [129]. Low selenium status has been associated with an increased risk for chronic diseases, such as cancer, type-2 diabetes, and cardiovascular disease [130,131].

Selenium can change the miRNA profile in an intestinal cell line (Caco-2), differentially downregulating 12 miRNAs under conditions of deficiency. The miRNAs that are most affected are miR-185, miR-625, miR-203, and miR-429. Pathway analysis identified arachidonic acid metabolism, glutathione metabolism, oxidative stress, positive acute phase response proteins, and respiration of mitochondria as selenium-sensitive pathways [18]. In a rat model of selenium deficiency, five miRNAs extracted from harvested heart (miR-374, miR-16, miR-199a-5p, miR-195 and miR-30e*) were identified as upregulated >5-fold in the deficiency group, compared to the selenium-supplemented group, and three were downregulated (miR-3571, miR-675 and miR-450a*). The upregulated miRNAs were involved in signal transduction, cell differentiation, and as a response to stress. Their wide range of actions may regulate cardiac function [132].

In humans, interventions in elderly males with selenium and coenzyme Q10 as supplements for four years led to substantial differences in 101 miRNAs. Most of these miRNAs play roles in cardiovascular disease processes, oxidative stress, and inflammatory activity, resulting in reduced cardiovascular mortality, better cardiac function, and less signs of inflammation and oxidative stress [133].

Another relevant mineral related to inflammatory processes is zinc, considered one of the most important trace elements in the body since it is the only metal that is a cofactor to more than 300 enzymes and is also required for the structural stability of a huge number of proteins [134]. Zinc deficiency causes overexpression of inflammatory genes and altered miRNA expression across several tissues in rats (skin, lung, pancreas, liver, prostate and peripheral blood mononuclear cells), particularly causing dysregulation of miR-31 and miR-21, associated with inflammation, as compared with zinc-sufficient animals [135]. In humans, a total of 20 miRNAs were shown to respond to a dietary zinc depletion regimen, this effect was reversed by subsequent zinc repletion. miR-204 and miR-296-5p presented the greatest down-regulation in response to dietary zinc depletion. It is of note that both of these miRNAs have been shown to suppress oncogene expression [136].

### 3.8. Vitamins

Several vitamins are suggested to modulate the immune system and prevent diseases. Vitamin D can regulate the transcription of miRNA genes through its active form, dihydroxy vitamin D (1,25(OH)2D), which binds to the vitamin D receptor (VDR) transcription factor, inducing miRNA maturation by regulating the genes involved in miRNA processing (such as Drosha, and Dicer) or miRNA stability [137,138].

Vitamin D down-regulates inflammation by targeting miRNAs, such as miR-155, which negatively correlates with VDR. The miR-155 level is correlated with the expression of suppressor of SOCS1 protein level in liver and PBMC in patients with primary biliary cholangitis [139]. Furthermore, vitamin D deactivates NF-κB signaling, via p65 and IκB phosphorylation inhibition in murine adipocytes by decreasing the expression of miR-146a, miR-150, and miR-155 [140]. VDR activators (such as calcitriol and paricalcitol) can inhibit the expressions of miR-29b and miR-30c in nephrectomized rats. These miRNAs seem to act on COL1A1, MMP-2, and CTGF expression, attenuating cardiac fibrosis [141]. In humans with moderate chronic kidney disease, paricalcitol treatment for 12 weeks reduces cytokines and microRNAs involved in atherosclerosis and inflammation [142].

In patients with primary prostate cancer, miR-100 and miR-125b levels were significantly lower in tumor tissue than in benign prostate tumors. These miRNAs have known tumor suppressor activities, and vitamin D treatment has been shown to increase their levels and decrease their targets (E2F3 and PLK1) in a dose-dependent manner in primary prostatic epithelial cells. These findings suggest that vitamin D supplementation augments tumor suppressive miRNAs in patient prostate tissue [143].

Vitamin A derivatives (retinoic acid isoforms) also regulate the expression of various genes under both physiological and pathological conditions [144]. In mouse embryonic stem cells, treatment with retinoic acid significantly upregulated 31 miRNAs and downregulated 175 miRNAs. Two of the downregulated miRNAs (miR-200b and miR-200c) significantly increased the expression of two pluripotent genes (Oct4 and Nanog), which are relevant to the development and are a key determining factor of the epithelial phenotype [145].

Retinoic acid (RA) significantly increases miR-10a expression as well the Retinoic Acid Receptor Beta (RARβ) in breast cancer cell lines (T47D and SK-BR-3). Loss of miR-10a and RARβ expression are associated with breast cancer since they are related to tumor suppressor in breast tissue specimens [146]. In addition, RA promotes iT_reg_ cells (inducible regulatory T cells) differentiation and induces miR-10a in non-T cells {125}. The increased expression of miR-10a in T_reg_ cells by RA treatment results in the preserved phenotype of T_reg_ cells by targeting and constraining transcription-factor pathways that promote alternative fates [147].

Table 1 lists some studies cited above and the principal miRNAs that are modulated by the respective nutrient and bioactive compound reviewed here (resveratrol, saturated fatty acids, unsaturated fatty acids, curcumin, quercetin, catechins, minerals, and vitamins).

## 4. Food-Derived microRNAs

As mentioned above, nutrients can modulate miRNA concentrations, conferring an important mechanism for the control of gene expression. In 2012, Zhang et al. [149] showed that food-derived miRNA could be absorbed in the intestine, demonstrating a new possible role of food beyond minerals, vitamins, macronutrients and bioactive compounds. This study detected rice miRNAs that were present in mammal plasma and tissues and detected the low-density lipoprotein receptor adapter protein (LDLRAP) as a target for plant-derived miRNA (MIR-168a), present at a high level in human serum. Furthermore, the elevation of LDL levels was detected in a mouse experiment, and was partly associated with a decrease in LDLRAP in the liver by MIR-168a [149]. Chen et al. [150] also identified 132 plant-derived miRNAs (miR-166a and miR-159a were the most abundant) in the blood of mice fed on 10 g of rapeseed bee pollen/kg of body weight. In humans, Baier et al. [151] evaluated the plasma samples of four healthy adults that consumed broccoli, and observed no change in broccoli-derived miR (miR-824 and miR-167a) in plasma at 4 h after 102 grams of broccolis intake.

Snow et al. [152] reported contrasting results from human and mice experiments. After the collection of blood samples from 10 healthy men volunteers who had a typical Western Diet, authors investigated three high expression fruit-miRNAs (miR-156a, miR-159a, miR-169a), but did not find any of these miRs in the samples. Similarly, in B57Bl6 wild-type miR-21 knockout male mice (8–12 weeks of age) fed on an animal lard diet replete in miR-21 over four weeks, no increase in the concentration of this miR was observed. The authors concluded that humans and mice are unable to maintain steady-state levels of plant and animal miRNAs in the circulation after the intake of a typical Western Diet or a chow specific diet. Thus, to increase plant-miR concentrations in animal cells, food consumption must be substantially greater than normal dietary circumstances [152].

Micó et al. [153] investigated plant-derived microRNAs in beer and extra virgin olive oil, but did not find any miRNA in these foods. These authors then administered 40 mL of extra virgin olive oil to five healthy volunteers (25–35 years old) and collected blood at baseline and 2 h after the intake; however, no plant-derived miRs were detected [153].

Since the bioavailability of food-derived microRNAs has been questioned, Philip et al. [154] studied miR-166, -167, -168 bioavailability after processing and cooking soybeans and brown rice and their survival after artificial digestion. When comparing raw and cooked beans and rice, cooked food present higher miRNA levels than raw food, suggesting a possible increase in bioavailability due to the disintegration of the cell wall structure of the plant, while the cooking water retains some of the food miRNAs. Additionally, authors concluded that all three microRNAs were stable after artificial digestion. Similarly, milk-derived miRNAs are suggested to be more resistant to enzymatic digestion, due to exosomal/vesicle transport [155]. Benmoussa et al. [156] assessed commercial milk-derived miRNA (bta-miR-223 and bta-miR 125b) resistance to the simulation of digestion via the computational control of an in vitro gastrointestinal model and quantified exosomal miRs. At 30 min after digestion, 64% of miR-223 was degraded, while 23% of miR-125 was degraded, demonstrating the distinct digestion kinetics of these two miRs. However, above 10^9^ miR copies were found in the small intestine, demonstrating the resistance of these miRs to the digestion process. Of these, more than 90% of milk-derived miRNA were not associated with exosomes, but authors suggested that a possible variation in vesicle size could occur, which was not detected for this group.

A study of animal breast milk-derived miRNA did not observe miR-375 or miR-200c/141 absorption after breastmilk intake by newborn mice that were knocked out for these two microRNAs. The miRNA concentrations did not change significantly in the intestinal epithelium (where a possible miRNA degradation was verified), blood, or spleen [157]. In contrast to these results, in a randomized crossover study, five healthy adults (26–49 years old) were found to absorb miR-29a and miR-200c after consumption of 3 milk doses, which were calculated by body water composition. Plasma samples were collected at 0, 1, 3, 6, 9, and 24 h after ingestion [151].

As mentioned above, food-derived miRNA represents a contradictory theme, in which still lacks studies that prove the function of these microRNAs interindividual and interspecies, as well as there is still a shortage of studies that robustly demonstrate whether plant and animal food derived microRNAs are absorbed by mammals [158,159]. Some conditions instigate authors to question the importance of miRNA like possible epigenetic communicators These conditions corresponding to a sequence conservation of microRNAs interspecies, presence and high stability in body fluids and extracellular vesicles transport. Whereas, the absorption of animal-derived microRNA can be absorbed in significant amounts, e.g., from milk, the absorption of plant-derived microRNAs appeared to be negligible in some studies. However, as the structure of animal-derived microRNA is common in the animal kingdom, it is much more difficult to distinguish the various sources of microRNA. [160]. Diverse reviews debate about this theme, but few experimental studies are found. Bipartition of authors’ opinion is clear and those who consider microRNAs as epigenetic cross-kingdom communicators, see these non-coding RNA as alternative human disease therapy [161,162].

## 5. Conclusions

The inflammatory response that is metabolically induced by excess nutrients is an important causative factor of chronic diseases such as obesity, type 2 diabetes, cardiovascular disease, and cancer. Few studies are available in the literature that shows the effect of nutrients and bioactive compounds that regulate human miRNAs in different metabolic situations. Much of the available studies are conducted in vitro and in animals. In humans, nutrition science studies may helpto identify new biomarkers and other possible mechanisms of action of certain dietary compounds, with a view to understanding how nutrients and bioactive compounds can control these small non-coding RNAs and regulate physiological mechanisms.

## Figures and Tables

**Table 1:** MicroRNAs regulated by nutrients, according to tissue, model evaluated and target prediction.

Nutrient	microRNA	Regulation and Tissue	Model	Targets	Reference
EPA/DHA	miR-146a-5p, -7b-5p, -155-5p, -125a-3p	↑ cells	Macrophage cell line (RAW264.7) and epithelial cells (TIME)	-	Roessler et al. [106]
EPA	miR-221	↓ cells	Human endothelial progenitor cells (hEPC)	-	Chiu et al. [115]
Oleic acid	miR-21	↑ cells	Hepatocytes	PTEN	Vinciguerra et al. [16]
Resveratrol	miR-20a-5p	↓ plasma	Human (♀)	Ikk	Li et al. [89]
	miR-663	↑ cells	Human monocytes (THP-1)	JunB; JunD	Tili et al. [15]
	miR-155	↓ cells	Human monocytes (THP-1)	-	Tili et al. [15]
	miR-Let7A	↑ cells	Human monocytes (THP-1)	-	Song et al. [88]
	miR-21	↓ cells	Human glioblastoma cells (U251)	-	Li et al. [89]
	miR-146	↓ cells	Macrophage cell line (RAW264.7)	Nfr2	Bigagli et al. [90]
	miR-21, miR-181b, miR-663, miR-30c2	↑ plasma	Human (♀)	-	Tomé-Carneiro et al. [91]
	miR-155, miR-34a	↓ plasma	Human (♀)	-	Tomé-Carneiro et al. [91]
Curcumin	miR-155-5p	↓ cells	Macrophage cell line RAW264.7	SOCS1; SHIP1	Ma et al. [119]
		↓ liver and kidney	Male C57BL/6 mice		
	miR181b	↑ cells	Breast cancer cell (MDA-MB-231)	CXCL-1; CXCL-2	Kronski et al. [20]
	miR-17-5p	↑ cells	3T3-L1	Tcf7l2	Tian et al. [121]
Epigallocatechin gallate (EGCG)	miR-let-7b	↑ cells	Human melanoma cells (Mewo and A375)	HMGA2; PKA; PP2A	Yamada et al. [128]
	miR-16	↑ cells	Human hepatocellular carcinoma cells (HepG2)	Bcl-2	Tsang et al. [148]
	miR-140-3p	↑ cells	Human chondrocytes from osteoarthritic cartilage	ADAMTS5	Rasheed et al. [19]
Quercetin	miR-122	↑ liver	Female C57BL/6 mice	AOAH	Boesch-Saadatmandi et al. [123]
	miR-125b	↑ liver	Female C57BL/6 mice	TNF-α	Boesch-Saadatmandi et al. [123]
	miR-155	↓ cells	Macrophage cell line RAW264.7	TNF-α	Boesch-Saadatmandi et al. [124]
Selenium	miR-185, miR-625, miR-203, miR-429	↓ cells	Human intestinal cell line (Caco-2)	-	Maciel-Dominguez et al. [18]
	miR-374, miR-16, miR-199a-5p, miR-195, miR-30e*	↑ heart	Rats with selenium deficiency (miRNAs extracted from harvested heart)	-	Xing et al. [132]
	miR-3571, miR-675, miR-450a*	↓ heart		-	Xing et al. [132]
Zinc	miR-31, miR-21	↑ esophagus and tongue	Rats with zinc deficiency	PPP2R2A; PDCD4	Alder et al. [135]
	miR-204, miR-296-5p	↓ serum	Young male humans on dietary zinc depletion regimen	-	Ryu et al. [136]
Vitamin D	miR-100	↑ cells	Primary prostatic epithelial cells	PLK1	Giangreco et al. [143]
	miR-125b	↑ cells		E2F3	Giangreco et al. [143]
	miR 432-5p, miR 495-3p, and miR 576-5p	↓ plasma	Patients with moderate chronic kidney disease	-	Mansouri et al. [142]
Vitamin A	miR-200b, miR-200c	↓ cells	Mouse embryonic stem cells	Oct4; Nanog	Zhang et al. [145]
	miR-10a	↑ cells	Breast cancer cell lines (T47D and SK-BR-3)	-	Khan et al. [146]
		↑ cells	Naturally occurring T_reg_ cells from Foxp3^EGFP^ mouse	Bcl-6; Ncor2	Takahashi et al. [147]

↓ downregulated; ↑ upregulated. EPA: eicosapentaenoic acid; DHA: docosahexaenoic acid; PTEN: phosphatase and tensin homolog; Ikk: inhibitor of nuclear factor kappa-B kinase; JunB, JunD: proteins from the Jun family; Nfr2: the transcription factor nuclear factor (erythroid-derived 2)-like 2; SOCS1: suppressor of cytokine signaling 1; SHIP1: phosphatidylinositol 3,4,5-trisphosphate 5-phosphatase 1 isoform a; CXCL: C-X-C motif chemokine ligand; Tcf7l2: transcription factor 7 like 2; HMGA2: high mobility group AT-hook 2; PKA: protein kinase A; PP2A: protein phosphatase 2A; Bcl-2: apoptosis regulator Bcl-2 beta isoform; ADAMTS5: disintegrin and metalloprotease with thrombospondindomains (aggrecanse-2); AOAH: acyloxyacyl hydrolase; TNF-α: tumor necrosis factor-alpha; PPP2R2A: protein phosphatase 2 regulatory subunit Balpha; PDCD4: programmed cell death 4; PLK1: polo like kinase 1; E2F3: transcription factor E2F3 isoform 2; Oct4: organic cation/carnitine transporter4; Nanog: Nanog homeobox protein; Bcl-6: B-cell lymphoma 6 protein; Ncor2: nuclear receptor corepressor 2.
